# Elemental Mercury Spills

**DOI:** 10.1289/ehp.7048

**Published:** 2005-09-29

**Authors:** Thomas A. Baughman

**Affiliations:** Environmental Toxicology Section, Illinois Department of Public Health, West Chicago, Illinois, USA

**Keywords:** cleanup, elemental mercury, health effects, mercury, prevention, remediation, spill, spill management

## Abstract

Sources of elemental mercury (Hg^0^) include old natural gas regulators, manometers, sphygmomanometers, thermometers, and thermostats. Causes of Hg^0^ spills include improper storage, container breakage, children playing with Hg^0^, the breakage of devices containing Hg^0^, and ritualistic use of Hg^0^. Inhalation is the primary exposure route for Hg^0^. Mercury released into the environment can enter lakes and streams, where bacteria convert it into methylmercury, which bioaccumulates in fish. Chronic exposure to Hg^0^ vapors can damage the kidneys and neurologic system. Short-term exposure to high levels of Hg^0^ vapors may cause lung damage, nausea, vomiting, diarrhea, increases in blood pressure or heart rate, skin rashes, and eye irritation, among other effects. Minimizing Hg^0^ dispersal is important after an Hg^0^ spill. Tracking by shoes or apparel or vacuuming can spread Hg^0^, increasing airborne concentrations and cleanup costs. The Illinois Department of Public Health’s response to an Hg^0^ spill depends on the size of the spill. Airborne concentrations after large spills are mapped with a mercury vapor analyzer (MVA). The cleanup begins with the spill site and any hot spots that were identified with the MVA. Hard surfaces can usually be cleaned, but contaminated porous items must be discarded. Leaving marginally contaminated items outdoors for a month or more during warm weather may dissipate the Hg^0^. After a cleanup, clearance sampling is conducted to determine if further cleanup is needed. The best way to prevent Hg^0^ spills is reduce its use.

Elemental mercury (Hg^0^), the silvery liquid most people associate with thermometers, is a liquid metal at room temperature. More than 13 times heavier than water, 1 tablespoon of Hg^0^ has a mass of about 150 g, and 1 L of Hg^0^ has a mass of about 13.5 kg. Hg^0^ evaporates slowly to produce vapors, the primary health concern.

## How Hg^0^ Spills Occur

Hg^0^ spills occur in many ways, often because of unnecessary or improper storage. Zeitz et al. (2002) reported the types and relative frequencies of 413 Hg^0^ spills reported from 14 states. Ninety-six percent of the spills occurred at fixed locations, and 4% of the spills were transportation related. Of the fixed-location spills, the most frequent locations were schools or universities (20.3%), private residences (16.7%), health care facilities (16.5%), public utilities (12.6%), and manufacturing facilities (10.0%).

People often keep Hg^0^ because they think it is valuable, but it is nearly worthless. Sometimes containers stored for years fall off shelves and break, or children find containers of Hg^0^ in a home or school and play with it. Zeitz et al. (2002) reported that in residences the most common causes of Hg^0^ spills were a spilled or dropped container (42%), children playing with Hg^0^ (32%), and equipment failure (17%). Schools often have containers with Hg^0^ (and other hazardous chemicals) that have been kept for many years. Zeitz et al. (2002) reported that children playing with Hg^0^ caused 46% of reported Hg^0^ spills in elementary and secondary schools. Other causes were a dropped or spilled container or instrument (18%), equipment failure (5%), and unknown (18%) (Zeitz et al. 2002).

**Figure 1:**
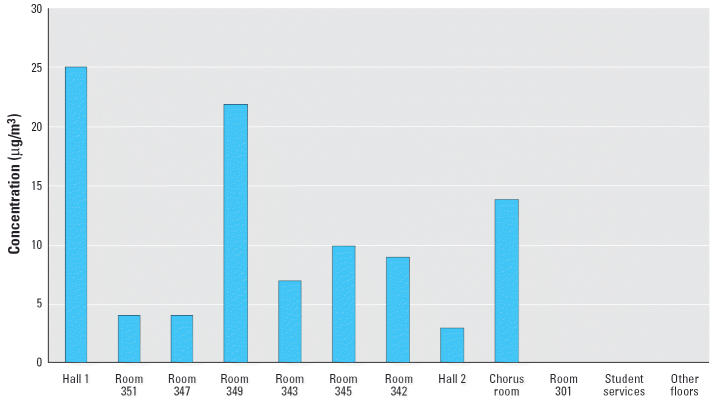
Airborne concentrations of Hg^0^ in a middle school after a 6th-grade student found a container with 4 lb Hg and played with its contents.

**Figure 2:**
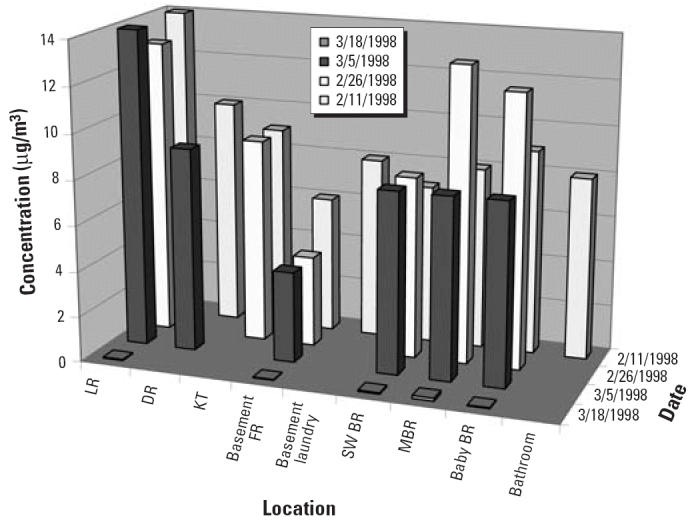
Airborne Hg^0^ concentrations in a home contaminated by a student who played with mercury at school. Abbreviations: BR, bedroom; DR, dining room; FR, family room; KT, kitchen; LR, living room; MBR, master bedroom; SW, southwestern.

Table 1 lists the sources and causes of Hg^0^ spills investigated by the West Chicago Regional Office of the Illinois Department of Public Health (IDPH). In one school that IDPH investigated, a sixth-grade student found a jar with about 4 lb Hg^0^ in an unlocked school cabinet. He threw beads of Hg^0^ into two hallways, and he gave Hg^0^ to friends, contaminating many areas of the school (Figure 1). The children thought they were having harmless fun. The school was unaware the Hg^0^ was present, although it was likely the Hg^0^ had been in the school since before 1973, when the building was used as a high school. The cleanup costs for the school district approached $200,000. Besides the school, IDPH sampled for airborne Hg^0^ in 11 homes of children who had played with the Hg^0^. Fortunately, only one home had enough Hg^0^ to require a cleanup (University of Wisconsin Extension 2003a; Figure 2).

Teenagers trespassing at abandoned industrial sites have found Hg^0^ and subsequently carried contamination into their homes and schools (George et al. 1996). Large Hg^0^ spills have been caused by broken blood pressure devices (containing about 150 g), other medical instruments (450 g), and old barometers (900 g). Old manometers, which are used to measure pressure, may contain Hg^0^. IDPH investigated one case involving a 12-kg spill from a manometer.

Some natural gas regulators made before 1961 contained Hg^0^, which was sometimes spilled when the regulators were removed. In Illinois, discovery of this problem triggered a large inspection and cleanup program by natural gas utility companies. One of these companies visually inspected about 300,000 homes. They sampled Hg^0^ vapor in 154,543 homes and found Hg^0^ contamination in 1,058 homes (slightly less than 1%).

Spills also have occurred when Hg^0^ is used as a folk remedy or during religious practices. Ingesting Hg^0^ is a folk remedy used by some cultures to treat alcoholism, colic, constipation, stomachache, or nervousness. Some Hispanic and Caribbean religious practices use Hg^0^ for good luck, for warding off evil spirits, or as a love potion. Hg^0^ is sometimes sprinkled in or around a car or crib, carried in an amulet or purse, burned in a candle, or mixed with bath or cleaning water. Most often, Hg^0^ is sold in small community shops known as *botanicas*. Two studies performed in Chicago and New York documented the sale of Hg^0^ in these shops. In Chicago, all 16 botanicas visited by the Chicago Department of Public Health sold Hg^0^. In New York City, > 90% of the botanicas visited sold Hg^0^ daily. Many buyers carried Hg^0^ in sealed containers, but almost one-third of the buyers sprinkled Hg^0^ in their homes [Chicago Department of Public Health 1997; U.S. Environmental Protection Agency (EPA) 2002].

## Exposure Routes, Persistence, and Health Effects of Hg^0^

Inhalation is the main route of concern because 80% of inhaled Hg^0^ is absorbed. Absorption of Hg^0^ after ingestion is low. Some exposure through skin can occur, especially if cuts are present. Because the body eliminates mercury slowly, cumulative exposure is important [Agency for Toxic Substances and Disease Registry (ATSDR) 1999].

After a large Hg^0^ spill, the hazard can persist for a long time. In the case of natural gas regulator spills, monitoring found elevated airborne Hg^0^ > 10 years after it was spilled. Several factors contribute to the persistence of Hg^0^. Spilled Hg^0^ forms small beads, which spread, making a thorough cleanup difficult. Hg^0^ tends to soak into building materials with time, and it generally cannot be removed from porous material, such as carpeting, clothing, drywall, fiberboard, unfinished wood, and upholstered furniture.

Children are more sensitive than adults to mercury. Four factors contribute to this: *a*) Hg^0^ vapors are heavy and settle, making concentrations higher at floor level, where young children play; *b*) the blood–brain barrier of children is less able to keep mercury out of the brain; *c*) the respiration rate of children is higher than that of adults, so children inhale more Hg^0^ at a given concentration than do adults; and *d*) the nervous system of children is still developing (ATSDR 1999, 2002, 2004).

At room temperature, short-term (acute) health effects from Hg^0^ are infrequent; however, heating Hg^0^ increases its evaporation. This can cause very high airborne concentrations and severe acute health effects. In Michigan, four adults melted down tooth fillings to recover the silver. However, these fillings contained about 50% mercury. After 1 day, all four people developed difficulty breathing. Despite medical care, all four patients died within 11–24 days. The home where the fillings were melted down was so contaminated that it had to be destroyed (Taueg et al. 1991).

At room temperature, long-term (chronic) exposure may cause adverse health effects. Chronic exposure of 1 month or more to low levels of Hg^0^ can cause nervous system and kidney damage. Neurologic symptoms of mercury poisoning include decreased nerve impulse conduction, decreased motor skills (e.g., finger tapping and hand–eye coordination), irritability, poor concentration, shyness, tremors (initially affecting the hands and sometimes spreading to other parts of the body), incoordination (e.g., difficulty walking), and short-term memory loss. The motor skill effects may be reversible, but short-term memory loss may be permanent. Other symptoms may include abdominal cramps, diarrhea, eye irritation, nausea, skin rashes, and weight loss. Children may experience acrodynia, which is characterized by pink-colored palms and soles of the feet, excessive sweating, flushing, itching, joint pain, rashes, swelling, weakness, irritability, worry, and trouble sleeping (ATSDR 1999). One study associated chronic exposure of 10–40 μg/m^3^ with neurologic effects in children (Taueg et al. 1991), but the threshold for effects is uncertain. In workers, one study associated chronic exposure of 26 μg/m^3^ with neurologic effects. However, some studies reported no neurologic effects at slightly higher concentrations (ATSDR 1999).

Misdiagnosis of mercury poisoning, often as a psychological disorder, is a common problem. Before the correct diagnoses, patients often worsen after returning to their contaminated homes (Centers for Disease Control and Prevention 1990; Florentine and Sanfilippo 1991; George et al. 1996; Rennie et al. 1999; Taueg et al. 1991; Tominak et al. 2002; U.S. EPA 2002).

In almost all cases, IDPH was called soon after a spill, so significant exposure did not occur. In five old spills, Hg^0^ levels of concern occurred only in little-used areas of basements (four spills) or a garage (one spill), so significant exposure did not occur. In homes affected by spills from old natural gas regulators, the spills also mainly affected basements, minimizing exposure.

To date, IDPH has had only one spill in which children experienced adverse health effects. At the end of the 1993 school year, children were helping move a school chemistry laboratory to another room. One child took home a container with 1 lb Hg^0^. During the summer, this child and siblings played with Hg^0^ on table-tops and carpeting and played “tin man” by applying Hg^0^ to their skin. Symptoms appeared about 1 month later, when two of the children were hospitalized (Table 2). Doctors initially suspected thrombocytopenia, possibly caused by lupus or an infection. The doctors performed many tests for bacterial and viral infectious agents and autoimmune problems, all with negative results. The 10-year-old child almost died twice from respiratory arrest, and he underwent an emergency (and unnecessary) splenectomy, as possible treatment for thrombocytopenia. The correct diagnosis was not made until 3 months after symptoms began, when the children confessed to playing with Hg^0^. Four months after the children took the Hg^0^, airborne Hg^0^ levels in the home measured with Hopcalite (SKC, Inc., Eighty Four, PA) tubes were very high—110 μg/m^3^ in the living room and 140 μg/m^3^ in the basement family room.

## Regulatory Standards and Advisories for Hg^0^

The occupational exposure limit set by the U.S. Occupational Safety Health Administration is 100 μg/m^3^ as a time-weighted average (TWA) for 8 hr/day, 5 days/week (NIOSH 1997). The American Conference of Governmental and Industrial Hygienists (ACGIH) recommends a maximum Hg^0^ concentration of 25 μg/m^3^ as a TWA for the same exposure duration (ACGIH 1994). Because children are more sensitive than adults to mercury, occupational standards do not apply to them. For Hg^0^, the recommended limit for continual habitation by children is 0.2 μg/m^3^, according to the ATSDR (1999). However, this concentration is very hard to achieve after an Hg^0^ cleanup. For the natural gas regulator spills, the ATSDR and U.S. EPA worked with IDPH to develop suggested action levels for mercury vapors, 1 μg/m^3^ for clearance and a home evacuation level of 10 μg/m^3^ in living areas (ATSDR 2000).

## Response to Small Hg^0^ Spills from a Broken Thermometer or Thermostat

IDPH staff receive many calls about broken thermometers. After a broken thermometer incident, IDPH, using a mercury vapor analyzer, has never found airborne Hg^0^ concentrations > 1 μg/m^3^. Before September 2000, IDPH used a Jerome 431-X mercury vapor analyzer (Arizona Instrument, Tempe, AZ) for airborne Hg^0^ measurements. Since then, IDPH uses a Lumex RA915 mercury vapor analyzer (OhioLumex Co., Twinsburg, OH).

Once, a mother accidentally dropped a thermometer down a heating duct. Although heat increases the evaporation of Hg^0^, airborne Hg^0^ was still < 1 μg/m^3^. In another investigation involving a broken thermostat, IDPH found Hg^0^ vapor levels slightly > 1 μg/m^3^ directly above a bead of Hg^0^ on the floor. However, concentrations were < 1 μg/m^3^ a few feet away, so significant exposure would not occur. Consequently, air monitoring is probably not needed after a thermometer or thermostat is broken. Instead, IDPH informs people to pick up visible drops with masking tape or a medicine dropper, ventilate the room and avoid vacuuming the spill area for 2 weeks.

## Responding to Large Hg^0^ Spills

IDPH conservatively classifies anything larger than a broken fever thermometer or thermostat as a large spill. In the case of a large Hg^0^ spill, measures to reduce the spread of contamination are vital. However, homeowners or janitorial staff should not attempt to clean up a large Hg^0^ spill. Instead, a professional hazardous waste cleanup company, the state health department, or U.S. EPA should be contacted. A hazardous waste cleanup firm cleaning a residence or school should be familiar with residential cleanups and the ATSDR/U.S. EPA action levels. In one case, a firm unfamiliar with the ATSDR/U.S. EPA action levels did an inadequate cleanup of a school using only a special vacuum equipped with a filter to contain Hg^0^. Further cleanup was required.

After a large spill, IDPH recommends family members leave their home or apartment, particularly if young children or pregnant women are present, and will refer the family to an occupational physician familiar with mercury poisoning to monitor exposure. Several tests will show the amount of mercury in the body. If an acute exposure to Hg^0^ has occurred, a blood analysis will show mercury levels if performed within 3 days after the exposure. In adults, the background concentration of mercury is normally < 1.5 μg/dL blood (IDPH 2004b).

If a chronic, low-level exposure is suspected, a 24-hr urine specimen is the best measure of Hg^0^. If a 24-hr specimen is not possible, the first morning void is the best substitute. For adults, the normal background concentration of mercury in urine is < 20 μg/L (IDPH 2004b).

The extent and cost of an Hg^0^ cleanup often depend more on the spread of contamination than on the actual amount of Hg^0^ spilled. So before the arrival of a qualified cleanup crew, IDPH recommends a number of actions to reduce the risk of further contamination. Shoes can easily track Hg^0^, so removing them before leaving a room where a spill has occurred can prevent this. (IDPH staff use nonslip, chemically protective disposable booties to avoid contaminating shoes). Hg^0^ also can be spread throughout a home if it is on a person’s clothes. Additional methods to reduce the spread of Hg^0^ include limiting the number of people entering a home, covering the affected floor with plastic, and ensuring that any investigation of the spill moves from the least contaminated area to the most contaminated area before exiting through the nearest door. The following examples that IDPH has investigated show how important it is to minimize the spread of contamination (University of Wisconsin Extension 2003b).

**Figure 3:**
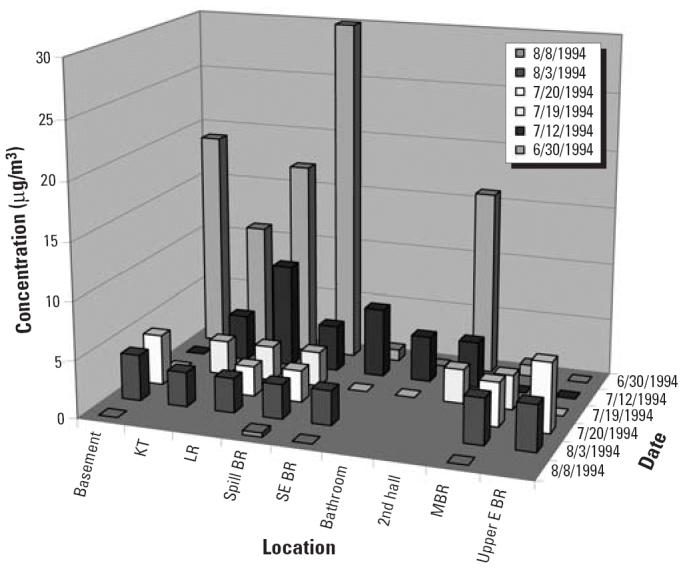
Airborne Hg^0^ concentrations after children broke a medical device with one pound of mercury. Abbreviations: BR, bedroom; E, eastern; KT, kitchen; LR, living room; MBR, master bedroom; SE, southeastern.

Several children were using a medical device, containing 1 lb Hg^0^, as a toy. In the course of their play, the children broke the device, splattering a crib, the wall, and the carpeting. The children’s father did nearly everything correctly: He put plastic on the floor of the room in which the spill occurred, changed shoes upon leaving the room, and kept his family out of the room until he could get them out of the home. However, he failed to check his children’s clothing after the device was broken. By the time the family left the home 2 hr after the spill, the children had spread Hg^0^ from their contaminated clothing to every room of the house (Figure 3). Initial concentrations in the spill room would have been higher, but a window was open. Subsequent increases in airborne Hg^0^ concentrations in other rooms suggested tracking by cleanup workers.

The family ultimately lost approximately 80% of the personal property in their home, including all the carpeting and most of the furniture. If the children (and cleanup workers) had not spread the Hg^0^, the cleanup would have been limited to just one room, thereby requiring less time and expense; the personal property loss would have been minimized, and the family could have returned home much sooner. In some cases where Hg^0^ is spread throughout a home, the cleanup costs can exceed the value of the home. This example underscores the importance of minimizing the spread of Hg^0^ after a spill. Also, never assume that a child who has been playing with Hg^0^ is uncontaminated.

**Figure 4:**
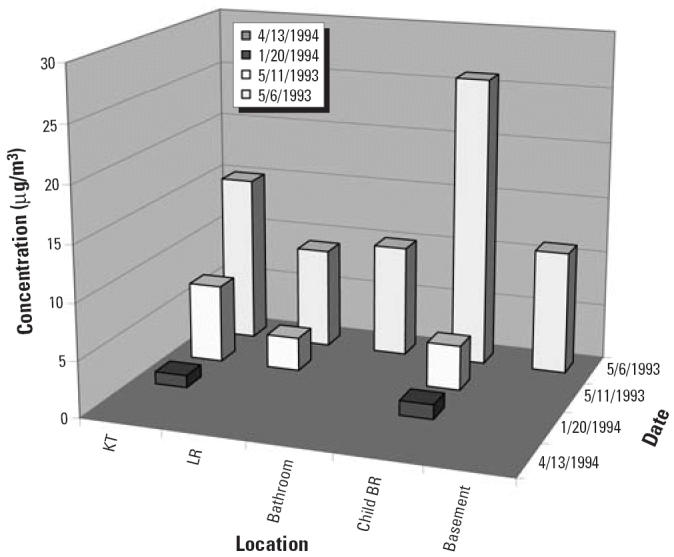
Airborne Hg^0^ concentrations after vacuuming. Abbreviations: BR, bedroom; KT, kitchen; LR, living room.

A second example illustrates the danger of using a conventional vacuum to clean up Hg^0^ (Figure 4). Conventional vacuuming heats the Hg^0^ and blows it into the air, spreading fine droplets and increasing airborne concentrations. In addition, the vacuum becomes permanently contaminated and must be discarded. In this particular spill, a jar containing 13 lb Hg^0^ fell off a kitchen pantry shelf and broke on the kitchen carpeting. The homeowner called the local fire department. Fire department personnel, rather than contacting a local hazardous materials team, vacuumed the Hg^0^.

Several subsequent cleanup attempts failed because the vacuuming had contaminated all surfaces in the kitchen. Ultimately, the entire kitchen had to be gutted. The ceiling, floor, and walls had to be removed, and all appliances and cabinets had to be discarded. Fortunately for the homeowner, two closed doors protected the rest of the home from serious contamination. If the entire house had been contaminated, cleanup costs could have exceeded the value of the home.

**Figure 5:**
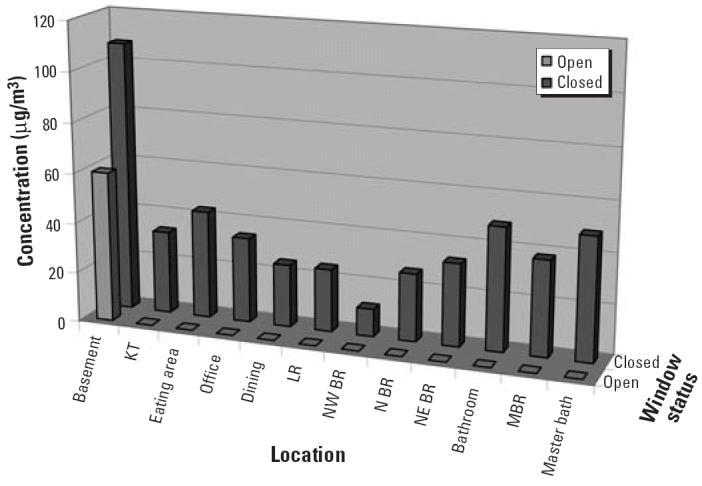
Effect of open windows on airborne mercury concentrations. Abbreviations: BR, bedroom; KT, kitchen; LR, living room; MBR, master bedroom; N, northern; NE, northeastern; NW, northwestern.

Factors affecting airborne Hg^0^ concentrations include not only the amount spilled but also ventilation, temperature, and the surface area of the Hg^0^ droplets (affected by dispersal). Consequently, the extent of airborne Hg^0^ concentration is hard to predict, making monitoring necessary. After taking measures to reduce the spread of contamination, IDPH uses monitoring instruments to assess airborne concentrations and the extent of contamination. The accuracy of the measurements depends on the instrument used and, with some instruments, can be affected by interferences from other chemicals present in the air (e.g., ammonia, cat urine, chlorine bleach, tobacco smoke). Air sampling with Hopcalite absorbent tubes and sampling pumps can be done for clearance sampling after a cleanup has been performed, but they require laboratory analysis. The Lumex mercury vapor analyzer provides real-time results and accuracy comparable with that of Hopcalite tubes. Hg^0^ evaporates slowly, so for meaningful results, windows should be closed at least overnight and preferably for 24 hr before any measurement is taken. In one case, a contractor measured airborne Hg^0^ concentrations in a home with windows opened, and they found Hg^0^ only in the basement. IDPH checked the home after windows were closed overnight, and airborne Hg^0^ concentrations throughout the home exceeded 10 μg/m^3^ (Figure 5).

Usually, cleanup of a spill site must be done before it can be determined whether airborne Hg^0^ away from the spill site is from the tracking of Hg^0^ or from the airborne dispersal of vapors. However, as an indirect indicator, the shoes of occupants can be tested for contamination. If the shoes are uncontaminated, airborne Hg^0^ contamination away from the spill site is likely due to airborne dispersion of vapors rather than tracking. If the shoes are contaminated, some tracking of the Hg^0^ probably occurred.

IDPH has found that, because of tracking, cleanup of a room where a spill has occurred does not usually reduce airborne Hg^0^ levels to < 1 μg/m^3^, and further efforts are needed. In instances where cleaning the floors of a home fails to reduce the Hg^0^ to acceptable levels, IDPH staff may turn their attention to household items, such as furniture, that may require decontamination or disposal.

This occurred in one case after a 1-lb Hg^0^ spill. After cleaning the floors of the home did not reduce Hg^0^ concentrations to acceptable levels, the resident placed all household contents in the back yard and wrapped or bagged them in plastic. IDPH staff then tested the bagged/wrapped contents for contamination. Using a Jerome mercury vapor analyzer, initial readings ranged from < 10 μg/m^3^ to > 20 μg/m^3^. Rather than recommending the immediate disposal of contaminated items, IDPH staff suggested leaving them outside for 1–2 months during the summer to see if some of the Hg^0^ would dissipate. This approach will work only in a warm location because Hg^0^ evaporation slows during cool or cold weather.

Bagged items with initial readings < 10 μg/m^3^ usually showed a decline to less than the 2-μg/m^3^ detection limit of a Jerome within a month. Follow-up readings for those items with findings of between 10 and 20 μg/m^3^ were different depending on the type of material. For hard-surfaced items, Hg^0^ levels generally declined to < 2 μg/m^3^; porous items, however, often remained contaminated. Items with initial readings > 20 μg/m^3^ showed little decline in contamination levels.

Testing of bagged items is a very sensitive method for detecting contamination. However, the airborne concentration of Hg^0^ a contaminated item may produce in the home is unknown. In one case, a child’s shoes gave a reading of 16 μg/m^3^ when placed in a plastic bag. Before this, however, a Jerome detected no Hg^0^ on the shoes, even when held just an inch from the shoes.

Another factor that may complicate successfully reducing indoor Hg^0^ contamination in air to 1 μg/m^3^ is the ability of Hg^0^ to seep into building components. For example, in the case of two old spills, airborne Hg^0^ concentrations in basements measured with Hopcalite tubes slightly exceeded the 1 μg/m^3^ threshold even after the areas had been cleaned and ventilated. However, upstairs Hg^0^ levels were < 1 μg/m^3^. Because both basements were infrequently used, the homeowners chose to live with the elevated levels in their basements rather than to remove the concrete floors. More recently, cleanup contractors have applied an epoxy sealant to such floors, effectively reducing airborne Hg^0^ concentrations.

## Step-by-Step Handling of a Large Hg^0^ Spill

**Figure 6:**
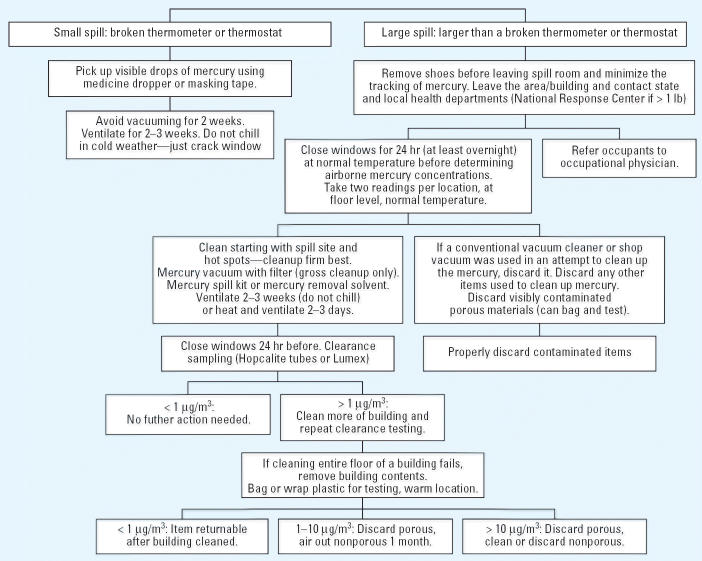
Handling Hg^0^ spills.

Figure 6 gives the procedures for cleaning an Hg^0^ spill. Hg^0^ can be cleaned up with sulfur or with a mercury spill kit (zinc). Both convert Hg^0^ into a less volatile form, but because powdered sulfur is very flammable, a mercury spill kit is the safer choice. The contents of the mercury spill kit are spread on the floor and worked into the cracks with a broom. Extended contact time is not necessary because zinc (and sulfur) reacts rapidly with any Hg^0^ that is present. The residue is then picked up with a broom and dustpan. Next, the surface should be washed with trisodium phosphate detergent and water. Some cleanup firms use nitric acid or an Hg^0^ removal solvent instead of a mercury spill kit. Hazardous waste cleanup firms often use vacuums with special Hg^0^ filters that pick up gross contamination. However, further cleanup generally is needed to reach the ATSDR/U.S. EPA clearance level. Contaminated hard surfaces—for example, linoleum, hardwood floors with a good finish, metal, plastic, or tile—are usually cleaned rather easily. Porous items, such as carpeting, clothing, fiberboard, unfinished wood, and upholstered furniture, that become contaminated generally cannot be cleaned and must be discarded.

## Hg^0^ Disposal

The Illinois Environmental Protection Agency periodically has household hazardous waste collections and also sponsors permanent collection facilities where residents can take Hg^0^ (and other chemicals) for free disposal (Appendix 1). Companies and schools must pay for disposal. Illinois has had pilot programs to assist schools in getting rid of mercury, but not on a permanent or statewide basis. For other states, contact the state environmental protection agency or the U.S. EPA regional office.

## Preventing Hg^0^ Spills

The best way to prevent spills is to keep Hg^0^ out of the home, school, or workplace. The U.S. EPA (1999) has recommended that Hg^0^ no longer be used for blood pressure and other medical devices, barometers, manometers, thermometers, or thermostats. The agency advises that these devices be replaced with Hg^0^-free alternatives, which are just as accurate and similar in cost. After one Hg^0^ spill in a nursing home, the Hg^0^ sphygmomanometer manufacturer told the facility not to replace their sphygmomanometers with nonmercury sphygmomanometers because the nonmercury devices were less accurate. However, several studies have shown that non-Hg^0^ blood pressure devices are equally accurate when calibrated annually, as recommended (Canzanello et al. 2001; Rouse and Marshall 2001; Yarows and Qian 2001).

Health care workers, the public, and school personnel should be educated about the hazards of Hg^0^, the availability of alternatives, and the cost of Hg^0^ cleanup. They need to know that Hg^0^ is almost worthless, not a valuable substance to be kept in the home as an investment. To aid in this education, IDPH has developed a mercury website and several educational pamphlets (Appendix 1).

## Mercury in the Environment

When Hg^0^ is released from industry, schools, or homes, it can end up settling into lakes and streams, where bacteria change it into methylmercury, a more toxic form. Methylmercury accumulates in animals and can reach high concentrations in fish. In fact, 37 states, including Illinois, have issued warnings about eating certain fish because of mercury contamination (U.S. EPA 1999).

## Conclusions

The best way to prevent Hg^0^ spills is not to store Hg^0^ in the home, school, or workplace. Because large Hg^0^ spills may cause hazardous conditions, particularly for children, and may pollute the environment, it is not advisable for homeowners or janitorial staff to undertake the cleanup. Such cleanups, which can be expensive, are best done by hazardous waste firms that are qualified to perform this work. To reduce the chances of a spill occurring, alternatives to Hg^0^-containing devices (e.g., thermometers, barometers, manometers, and blood pressure and other medical devices) should be used in homes, schools, medical facilities, and workplaces. Such devices are widely available and comparable in cost and work equally well. The adoption of these recommendations depends, however, on an informed public. People need to be educated about the hazards of Hg^0^, the costs of cleaning it up, and the availability of Hg^0^-free products.

## Figures and Tables

**Table 1:** Sources of mercury for spills investigated by the IDPH, West Chicago regional office.

		School		Medical facility	
	Home^a^	Source	Potentially affected homes^b^	Source	Potentially affected homes^c^
Number	25	3	11	2	5
Container	4	2	1	0	0
Thermometer, thermostat switch	7	0	0	0	0
Barometer	3	0	0	0	0
Switch	1	0	0	0	0
Manometer	3	0	0	0	0
Pressure regulator	1	0	0	0	0
Medical devices^d^	2	0	0	2	5
Unknown	4	1	0	0	0
Vacuumed	6	0	0	1	0
Children caused	1	1	11	1	?
> 1 to 10 μg/m^3^	9	0	1	0	1
≥ 10 μg/m^3^	9	3	2	2	0

? uncertain; shoes of both mother and child contaminated, so both may have contributed to contamination.

a Homes with indoor spills; does not include homes potentially affected by spills in schools (via children), medical clinics (via patients), or outdoors (24 apartments tested near outdoor spill of unknown cause; no apartments contaminated).

b Homes of children who played with Hg^0^ in one school.

c Homes of patients of one medical clinic who had contaminated shoes; the pediatrician instructed a nurse to clean up the mercury with a DustBuster, and then he continued seeing patients.

d Includes sphygmomanometers (two) and dilators (one).

**Table 2:** Symptoms and urine Hg concentrations in residents of a home with a Hg spill in Illinois.

Person	Symptoms	Urine Hg (μg/L)
Mother	Unknown	438
Father	Unknown	320
10-year-old male	Unable to walk, seizures, rash, nausea, vomiting, fever, cough, rash, thrombocytopenia platelets, melanotic stool with bright red blood	1,270
12-year-old male	Unable to stand, nausea, vomiting, rash	586
15-year-old female	Unknown	968
17-year-old female	Low-grade fever, rash, vomiting, thrombocytopenia	1,348

**Appendix 1:** Mercury websites.

Dates and locations of Illinois household hazardous waste pickup days and the locations of Illinois permanent collection facilities
http://www.epa.state.il.us/land/hazardous-waste/household-haz-waste/index.html
Educational websites on mercury developed by IDPH
Mercury in schools website (IDPH 2004c)
http://www.idph.state.il.us/mercury/
Mercury in schools fact sheet (IDPH 2004d)
http://www.idph.state.il.us/envhealth/pdf/mercuryschool.pdf
Mercury fact sheet for health professionals (IDPH 2004b)
http://www.idph.state.il.us/envhealth/factsheets/mercuryhlthprof.htm
Mercury spill fact sheet (IDPH 2004a)
http://www.idph.state.il.us/envhealth/factsheets/mercuryspills.htm
